# Dual E627K and D701N mutations in the PB2 protein of A(H7N9) influenza virus increased its virulence in mammalian models

**DOI:** 10.1038/srep14170

**Published:** 2015-09-22

**Authors:** Wenfei Zhu, Long Li, Zhigang Yan, Tanhuan Gan, Lifeng Li, Rirong Chen, Ruidong Chen, Zuoyi Zheng, Wenshan Hong, Jia Wang, David K. Smith, Yi Guan, Huachen Zhu, Yuelong Shu

**Affiliations:** 1 National Institute for Viral Disease Control and Prevention, Chinese Centre for Disease Control and Prevention, Key Laboratory for Medical Virology, National Health and Family Planning Commission, Beijing; 2 Joint Influenza Research Centre, Shantou University Medical College, Shantou, Guangdong; 3 State Key Laboratory of Emerging Infectious Diseases/Centre of Influenza Research, School of Public Health, The University of Hong Kong, Hong Kong SAR

## Abstract

The ongoing avian H7N9 influenza outbreaks in China have caused significant human fatal cases and the virus is becoming established in poultry. Mutations with potential to increase mammalian adaptation have occurred in the polymerase basic protein 2 (PB2) and other viral genes. Here we found that dual 627K and 701N mutations could readily occur during transmission of the virus among ferrets via direct physical contact, and these mutations conferred higher polymerase activity and improved viral replication in mammalian cells, and enhanced virulence in mice. Special attention needs to be paid to patients with such mutations, as these may serve as an indicator of higher virus replication and increased pathogenicity.

Since the initial outbreak in Eastern China in 2013^1^, the novel avian influenza A (H7N9) virus has caused more than 600 human infections with a mortality rate of over 35%^2^. Infected chickens at live-poultry markets were identified as the source of human infections and the H7N9 virus now appears to be becoming established in China^3^,^4^. The occurrence of repeated outbreak waves has posed an ongoing potential pandemic threat to the world.

With the development of the H7N9 virus in the field, some amino acid mutations have occurred which are believed to be associated with host preference and specificity in mammalian species^3^,^4^. As residues at positions 627 and 701 of the polymerase basic protein 2 (PB2) are considered critical for the mammalian adaptation of avian influenza viruses^5^,^6^,^7^,^8^, several studies have independently shown that single E627K (glutamic acid to lysine) or D701N (aspartic acid to asparagine) mutations could increase polymerase activity and viral replication in mammalian cells and the pathogenicity of H7N9 viruses in the BALB/c mouse model^9^,^10^,^11^. Yet it is still unknown whether spontaneous emergence of dual D701N and E627K mutations can occur readily in infected mammals and whether these will synergistically enhance the virulence of H7N9 viruses.

Here we used a ferret transmission model and deep-sequencing of the viruses shed by ferrets experimentally exposed to the prototype H7N9 virus, A/Anhui/1/2013 (AH1, with 627K and 701D in PB2)^1^ and an early precursor-like virus, A/Shanghai/05/2013 (SH5, with 627E and 701D in PB2)^12^. Our findings show that both the E627K and D701N mutations occurred in ferrets that had direct contact with infected animals within a few days post-exposure. Dual mutations of 627K and 701N, introduced by reverse-genetic techniques in the PB2 gene, significantly enhanced polymerase activity and virus replication in both human cells and C57BL/6 mice, giving increased virulence to the H7N9 virus.

## Results

### Transmission of H7N9 viruses in ferrets

All ferrets directly inoculated with a 10^6^ median tissue culture infective dose (TCID_50_) of the wild type AH1 or SH5 viruses shed viruses through the nasal cavity for 6 days post-inoculation (dpi) with peak titers, which occurred at 2 dpi, ranged from 6.0 ∼ 6.3 log TCID_50_/ml for AH1 and from 5.3 ∼ 6.3 log TCID_50_/ml for SH5 (Table 1). In the AH1 transmission group, all physical and airborne exposed ferrets seroconverted, and viruses were detected in all physical contact animals and two out of three airborne exposed ferrets (Table 1). Although seroconversions were observed in all three physical contact ferrets exposed to SH5, virus was only detected in the nasal washes of one animal (SH5-PC3) and no airborne exposed ferrets seroconverted or shed virus. The SH5-PC3 ferret started to shed virus 24 hours post-exposure (hpe) and the virus shedding titers peaked at 4 days post-exposure (dpe, Table 1). Relatively high shedding titers (>3.5 to 5.8 log TCID_50_/ml) were maintained in SH5-PC3 till 8 dpe (Table 1).

### PB2-627K and -701N mutations occurred in ferrets exposed to H7N9 viruses

All ferrets that shed virus after exposure to the wild type AH1 virus retained the 627K residue in the PB2 gene but two of the three physical contact animals carried a mixed population at the 701 position, with the D701N mutation occurring at a frequency of 94.2% and 86.8% respectively on their peak shedding day (3 and 4 dpe respectively, Table 1). Neither of the two AH1 airborne exposed ferrets that shed virus possessed the D701N mutation even though they were paired with the contact exposed animals that did. In the SH5 physical contact ferret (SH5-PC3), all viruses shed at 4 dpe retained 627E and 701D, but at 8 dpe 30% of the viral sequences contained 627K and 1.1% had 701N (Table 1), while 0.2% (10/4,844 reads) had both mutations. Thus, human isolates of H7N9 viruses could readily acquire dual 627K and 701N mutations after transmission to and replication in physical contact ferrets.

### PB2 627K and 701N increased polymerase activity of the AH1 polymerase complex

**Figure 1:**
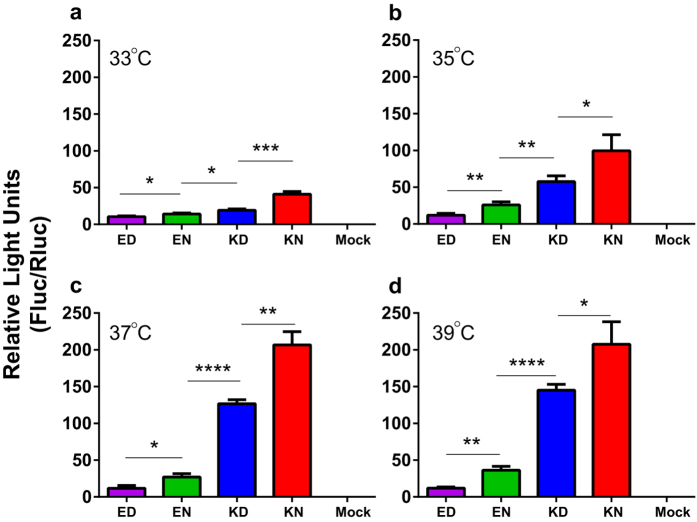
Polymerase activities of PB2 mutants in human cells cultured at different temperatures. 293T cells were transfected with a pFluc plasmid that expresses negative sense virus-like RNA encoding a firefly luciferase (Fluc) and a pRluc plasmid carrying the *Renilla* luciferase gene (Rluc) as an internal control (Promega). The 293T cells were also co-transfected with plasmids expressing the wild type (627K-701D, KD) and mutated PB2 (627E-701D, ED; 627K-701N, KN; or 627E-701N, EN), PB1, NP, and PA segments derived from the A/Anhui/1/2013(H7N9) virus. After culturing at 33 °C (**a**), 35 °C (**b**), 37 °C (**c**) and 39 °C (**d**) for 24 hours, cell lysate was used to measure Fluc and Rluc activity. Mean ± SD of triplicate experiments is shown. *p < 0.05; **p < 0.01; ***p < 0.001; ****p < 0.0001.

To investigate whether mutations at positions 627 and 701 in the PB2 protein could alter the activity of the RNA-dependent RNA polymerase, the expression levels of a luciferase reporter gene in minigeome-based assays in 293T cells were tested. A ribonucleoprotein (RNP) complex containing the PB2 627E and 701D (ED) residues consistently showed the lowest level of activity. The activity level increased from the PB2-627E+701N (EN) mutant to the wild type PB2-627K+701D (KD), with the mutant with 627K and 701N (KN) having the highest level of activity (Fig. 1). At all temperatures tested, PB2 627E mutants (ED and EN) reduced activity (0.27 - 0.92 fold) below that of the wild type virus (KD) while 701N (KN) increased activity by 0.43–1.15 fold (Fig. 1). Polymerase activity at 33 °C was generally lower than that at higher temperatures (*p *< 0.01), except for the mutant with PB2-627E+701D where the level of activity was not affected by temperature (Fig. 1). For mutants with 627K (KD or KN), polymerase activity at 35 °C was also significantly lower than that at the higher temperatures (*p *< 0.01).

### Dual PB2 627K and 701N mutations promoted H7N9 virus replication at 33 °C

**Figure 2:**
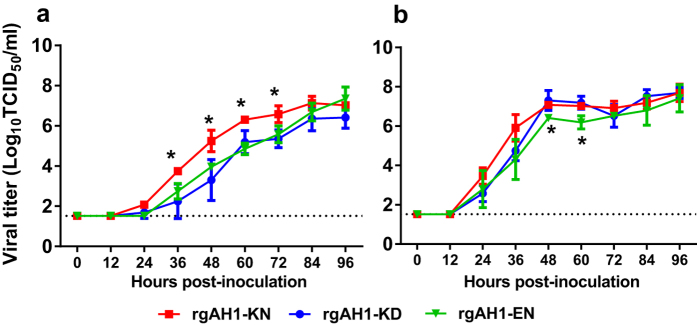
Replication kinetics of recombinant H7N9 viruses in human cells. Confluent monolayers of the cell lines were inoculated with recombinant viruses carrying mutations in the PB2 gene of the A/Anhui/1/2013 virus. Culture supernatants were harvested from human type II alveolar epithelial cells (A549) at 0, 12, 24, 36, 48, 60, 72, 84 and 96 hours post-inoculation (hpi) at 33 °C (**a**) and 37 °C (**b**), respectively. Virus titers were determined by TCID_50_ assays using MDCK cells. Results are presented as mean ± SD of three repeated experiments. rgAH1-KD (wild type virus carrying PB2-627K and 701D), rgAH1-KN (with PB2-627K and 701N), and rgAH1-EN (with PB2-627E and 701N). *p < 0.05.

To evaluate how the dual PB2 627K and 701N mutations contribute to the replication of the AH1 H7N9 virus, we compared the multicycle growth of the recombinant rgAH1-KD, -KN, and -EN viruses in A549 cells at 33 °C and 37 °C. These two temperatures were used to simulate the conditions in the upper and lower respiratory tracts of humans. Cells were inoculated at a multiplicity of infection (M.O.I.) of 0.001, and supernatant was sampled at various time points. At 33 °C rgAH1-KN exhibited significantly higher growth properties when compared to rgAH1-EN and the wild type rgAH1-KD virus at 36–72 hours post-inoculation (hpi, *p *< 0.05), with the average peak replication discrepancy occurring at 48 hpi (Fig. 2a). The replication kinetics of the rgAH1-EN virus was similar to that of wild type rgAH1-KD at all time points at 33 °C (*p* > 0.05, Fig. 2a). At 37 °C, rgAH1-KN and -KD replicated with highly similar kinetics within 96 hpi (*p* > 0.05), with rgAH1-KN showing marginally higher replication at 24–36 hpi (Fig. 2b). Both rgAH1-KN and KD replicated better than the rgAH1-EN mutant at 48–60 h at both temperatures (*p *< 0.05, Fig. 2).

### PB2 627K and 701N enhanced viral morbidity and mortality in mice

**Figure 3:**
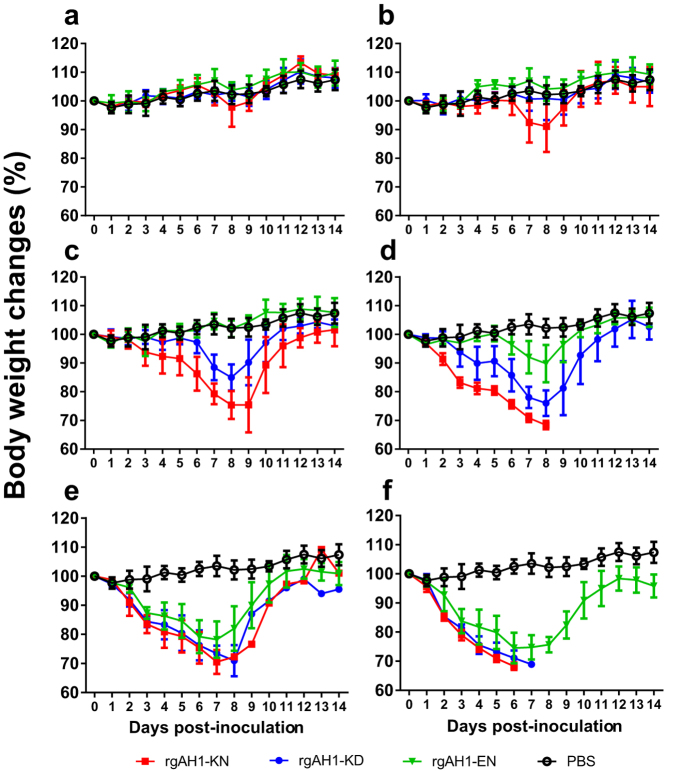
Infection of recombinant H7N9 viruses in mice. Eight- to ten-week-old female C57BL/6 mice (*n* = 5/group) were inoculated intranasally with 50 μl PBS containing 10^1^ (**a**), 10^2^ (**b**), 10^3^ (**c**), 10^4^ (**d**), 10^5^ (**e**) and 10^6^ TCID_50_ (**f**) of the recombinant A/Anhui/1/2013 (H7N9) viruses. Morbidity was assessed by body weight changes over a 14-day period post-inoculation. rgAH1-KD (wild type virus carrying PB2-627K and 701D), rgAH1-KN (with PB2-627K and 701N), and rgAH1-EN (with PB2-627E and 701N).

**Figure 4:**
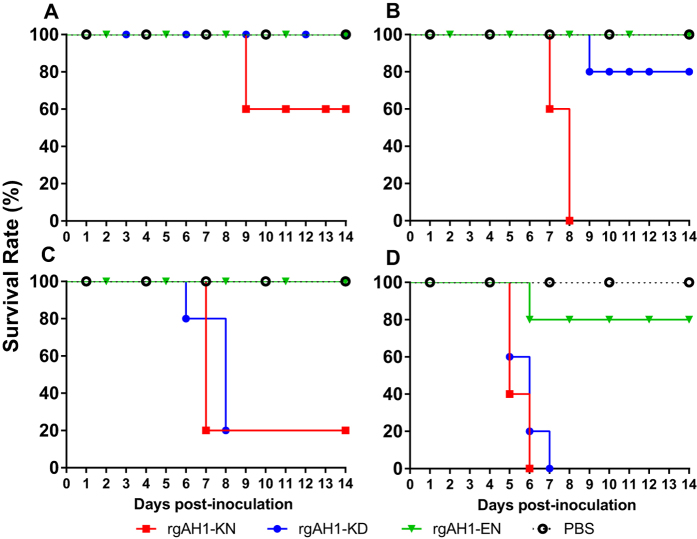
Mortality of recombinant H7N9 viruses in mice. Eight- to ten-week-old female C57BL/6 mice (*n* = 5/group) were inoculated intranasally with 50 μl PBS containing 10^1^, 10^2^ (data not shown as no mice died at these two doses), 10^3^ (**a**), 10^4^ (**b**), 10^5^ (**c**) and 10^6^ TCID_50_ (**d**) of the recombinant A/Anhui/1/2013 (H7N9) viruses. During the 14-day period post-inoculation, mice with a body weight loss of over 30% and those that died naturally were recorded as dead. rgAH1-KD (wild type virus carrying PB2-627K and 701D), rgAH1-KN (with PB2-627K and 701N), and rgAH1-EN (with PB2-627E and 701N).

To evaluate changes in the pathogenicity of H7N9 viruses conferred by mutations at the PB2 627 and 701 positions in mammals, ten-fold serially diluted recombinant viruses (rgAH1-KD, -KN, and –EN) were inoculated into groups of five mice. At inoculation doses of 10^1^ and 10^2^ TCID_50_, none of the mice died. The peak body weight loss at 8 dpi for a dose of 10^2^ TCID_50_ was less than 10% for the rgAH1-KN viruses and weight loss was not observed in other cases (Fig. 3a,b). At a dose of 10^3^ TCID50, two mice inoculated with rgAH1-KN died at 9 dpi, but no rgAH1-KD and rgAH1-EN inoculated mice died and no body weight loss was recorded in the rgAH1-EN inoculated mice (Figs 3c and 4a). At inoculation doses of 10^4^ TCID_50_ or above, significant body weight loss occurred in all three groups of mice, with the rgAH1-EN inoculated mice consistently showing the least weight changes and numbers of fatalities (Figs 3d-f and 4b-d). The MLD_50_ of the rgAH1-EN, rgAH-KD (wild type) and rgAH1-KN viruses were ≥10^6.3^, 10^4.5^, and 10^3.3^ TCID_50_ (Fig. 4), while the MID50 were 10^2.5^, 10^1.1^, and 10^0.9^ TCID_50_, respectively (Table 2). Overall, the virulence of the rgAH1-KN virus was the highest, followed in order by the rgAH1-KD (wild type), and rgAH1-EN viruses (Figs 3 and 4).

### Pathogenicity and tissue tropism of the H7N9 mutants in mice

**Figure 5:**
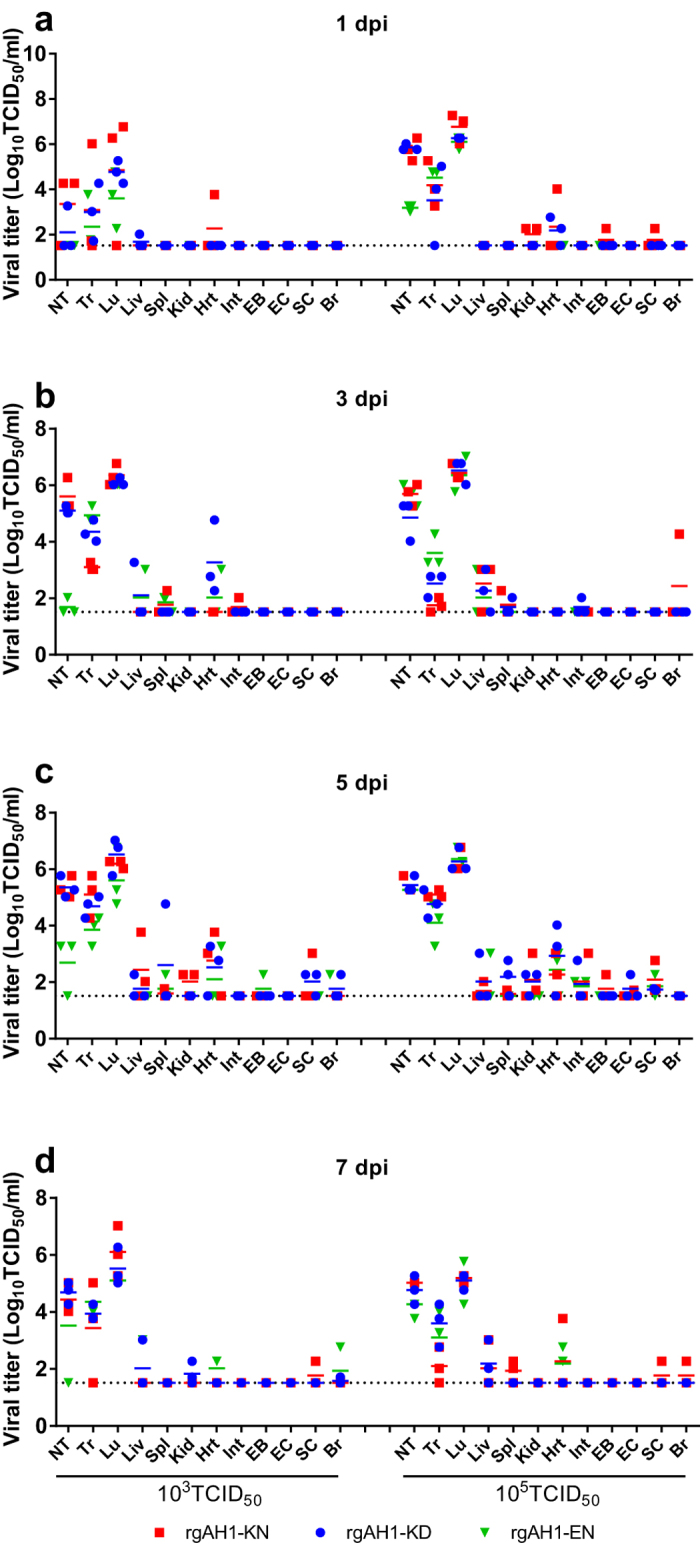
Replication of recombinant H7N9 viruses in the organs of mice. Eight- to ten-week-old female C57BL/6 mice (*n* = 3/group/time-point) were inoculated intranasally with 50 μl PBS containing 10^3^ TCID_50_ (left) or 10^5^ TCID_50_ (right) of the recombinant A/Anhui/1/2013 (H7N9) viruses. Animals were euthanized at 1 (**a**), 3 (**b**), 5 (**c**), and 7 (**d**) dpi. Tissues of each animal was homogenized in 1 ml of PBS and then clarified by centrifuge. Viral titers in the supernatant from homogenates were determined by TCID_50_ assays on MDCK cells. Results are presented as mean ± SD. rgAH1-KD (wild type virus carrying PB2-627K and 701D), rgAH1-KN (with PB2-627K and 701N), and rgAH1-EN (with PB2-627E and 701N). NT: Nasal turbinate; Tr: trachea, Lu: lung; Liv: liver; Spl: spleen; Kid: kidney; Hrt: heart; Int: intestine; EB: eye ball; EC, eye conjunctiva; SC: spinal cord; Br: brain.

**Figure 6:**
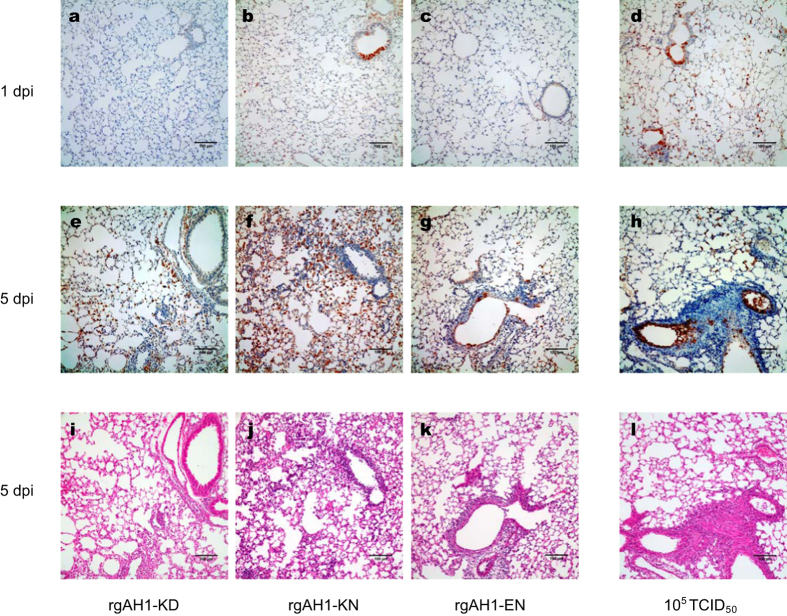
Virus infection and pathological changes in the lungs of mice inoculated with the recombinant H7N9 viruses. Eight- to ten-week-old female C57BL/6 mice (*n* = 3/group/time-point) were inoculated intranasally with 50 μl PBS containing 10^3^ TCID_50_ (**a**–**c**,**e**–**g**,**i**–**k**) or 10^5^ TCID_50_ (**d**,**h**,**l**) of the recombinant A/Anhui/1/2013 (H7N9) viruses. Animals were euthanized at 1 (**a**–**d**) and 5 (**e**–**l**) dpi, respectively, and the lung lobes were used for immunohistochemical staining with the anti-NP antibody (**a**–**h**) and standard hematoxylin and eosin (H&E, Sigma) staining (**i**–**l**). Slides were shown to represent the pathological changes and virus replication at different time points post-inoculation of rgAH1-KD (**a**,**e**,**i**), rgAH1-KN (**b**,**d**,**f**,**j**) and rgAH1-EN (**c**,**g**,**h**,**k**,**l**). rgAH1-KD (wild type virus carrying PB2-627K and 701D), rgAH1-KN (with PB2-627K and 701N) and rgAH1-EN (with PB2-627E and 701N). Scale bars indicate 100 μm.

Replication of the H7N9 recombinant viruses (rgAH1-KD, -KN, and –EN) in C57BL/6 mice was detected in both the upper and lower respiratory tracts (nasal turbinate, trachea and lungs) and occasionally in extrapulmonary organs (Fig. 5). Virus replication reached the highest titers in the nasal turbinates and lungs (Fig. 5), causing overt peribronchiolitis and pulmonaryalveolitis (Fig. 6). Prolonged virus replication in the respiratory tracts of the mice was observed for 7 dpi (Fig. 5d). Inflammation and virus replication in the bronchioloalveolar cells was more prominent at an inoculation dose of 5 log TCID_50_ than at 3 log TCID_50_ (Figs 5 and 6). Tissue tropism of the virus replication and the level of pathogenicity in the tissues were not significantly different with respect to the different PB2-627 and 701 mutants at an inoculation dose of 5 log TCID_50_ (Figs 5 and 6d [rgAH1-KN], h and l [rgAH1-EN]; data not shown for other tissues and viruses). At the 3 log TCID_50_ dose, slightly higher virus titers were detected in the lungs, nasal turbinates and a few extrapulmonary organs in the rgAH1-KN inoculated mice at 1 dpi (Figs 5a and 6a–c [lungs]), while significantly lower virus replication in the nasal turbinates and pathological changes in the lungs were observed in the rgAH1-EN inoculated mice (Figs 5 and 6).

## Discussion

Influenza A viruses can readily acquire mutations through virus replication in host cells, owing to the low fidelity of their RNA dependent polymerase^13^,^14^. At the beginning of the first outbreak wave of H7N9 in China, several potential mammalian adaptation markers had already appeared in H7N9 viruses that had infected humans, including PB2 627K and 701N^1^,^3^,^4^,^12^.

As of 4^th^ December 2014, all available H7N9 virus sequences from the poultry and environmental samples were found to exclusively carry PB2-627E while the majority (69.0%, 89/129) of the human isolates had a lysine mutation at this position (E627K), and a tree sparrow virus identified in Shanghai possessed both E and K^15^. An Aspartic acid to Asparagine mutation, relative to avian or environmental samples, was observed at position 701 in PB2 (D701N) in 8.5% (11/129) of human isolates. In January 2014, an H7N9 virus (A/Shanghai/PD-02/2014) carrying both the E627K and D701N mutations in the PB2 protein (GenBank accession number KJ549801) was isolated from a fatal human case, indicating that such dual mutations can occur naturally.

In this study, by using a ferret transmission model, we demonstrated that both the E627K and D701N mutations could occur within one passage in physical contact ferrets, and these two mutations could co-emerge in the same virus particle. The dual 627K and 701N mutations in the PB2 protein of AH1 viruses was associated with increased polymerase activity, better replication in human cells and enhanced virulence in mice. However, as the E627K and D701N mutations were not found in airborne exposed ferrets, this suggests they may not increase the airborne transmissibility of the viruses.

Structural studies of the C-terminal domain of PB2 show that residue 701 is located in the nuclear localization signal (NLS) and importin binding domain, and it might disrupt the salt bridge between 701D and 753R, thereby facilitating the unfolding of the NLS and its exposure to and binding of the importin molecules^16^,^17^,^18^,^19^. An increase in the nuclear accumulation of PB2 in mammalian cells could enhance the polymerase activity of an influenza virus carrying a 701N residue. PB2 proteins containing 627K or 627E possess nearly identical structures, but the electrostatic surface potential is more positively charged with 627K^16^,^17^,^20^. The large basic surface on the PB2 protein was assumed to be essential for efficient polymerase activity in human cells and may be involved in vRNP assembly or interactions with viral or host proteins and/or RNAs^17^,^19^,^20^.

The avian H7N9 influenza virus that emerged in 2013 has caused three outbreak waves in humans but relatively few viral sequences are available, given the large numbers of affected birds and humans. This paucity of information on the possible adaptations of H7N9 viruses within infected birds or patients makes the prediction of clinical outcomes difficult. Our study, together with previous reports^9^,^10^,^11^, highlights the importance of continuous monitoring of patients with H7N9 infection for mutations at positions 627 and 701 in the PB2 protein. Rapid genetic characterization of viruses recovered from the patients may better inform professionals about the prognosis of infections with H7N9 viruses. Surveillance in humans and other animals paying special attention to mutations in the virus that enhance its activity in mammals could lead to implementation of increased control measures and warnings to the public if indicators of a higher risk are identified.

## Methods

### Ethics statements

All experiments using mice and ferrets were conducted in compliance with approvals from the Committee on the Use of Live Animals in Teaching and Research (CULATR) of The University of Hong Kong (HKU CULATR 3264-14) and the Institutional Ethical Review Board (IERB) of Shantou University Medical College (Ref. No. SUMC2013-111 and SUMC2015-017). The methods were carried out in accordance with the approved guidelines of the International Council for Laboratory Animal Science and the University Policies “Animal Ethics and Welfare” and “Use of Animals in Research”.

### Biosafety and biosecurity statements

All manipulations involving H7N9 viruses were performed in biosafety level 3 (BSL3) containment facilities by trained staff with all necessary personal protective equipment, following the institutional Guidelines on Safety Policy & Management. Staff allowed to handle viruses and animals in the BSL-3 facilities must have completed BSL-3 training and got a license to conduct animal experiments. Active surveillance of body temperature and reporting of symptoms of influenza-like illness were required for researchers involved in this project. Individually ventilated cages and powered air purifying respirators were used for the animal experiments. Chemical and biosafety approvals were obtained from the Li Ka Shing Faculty of Medicine, HKU. The Faculty’s safety policy, management and practice are subject to constant review by the Faculty Safety Committee and its Safety Officers. Matters of dual use research are considered under the ethical approvals and safety approval procedures.

### Cells and viruses

Human embryonic kidney 293T and human type II alveolar epithelial A549 cells (ATCC CCL-185) were obtained from the American Type Culture Collection (ATCC) and maintained in Dulbecco’s modified Eagle’s medium (DMEM; Invitrogen, Carlsbad, CA, USA) supplemented with 10% fetal bovine serum (FBS; Invitrogen), glutamine (2 mM; Invitrogen), penicillin (100 units/ml), and streptomycin (100 μg/ml; Invitrogen). Madin-Darby canine kidney (MDCK, from ATCC) cells were maintained in minimum essential medium (MEM; Invitrogen, Carlsbad, CA, USA), supplemented with 10% FBS, penicillin (100 units/ml), and streptomycin (100 μg/ml; Invitrogen). All cells were incubated in a humidified atmosphere of 5% CO_2_ at 37 °C. Two human H7N9 virus isolates^1^,^12^, A/Anhui/1/2013 (AH1) and A/Shanghai/05/2013 (SH5), were passaged using 9- to 10-day-old embryonated chicken eggs. Viral titrations were determined using the tissue culture infectious dose affecting 50% of the cells (TCID_50_) on MDCK cells.

### Plasmids and mutagenesis

Viral RNAs of the A/Anhui/1/2013 (H7N9) influenza virus were extracted and reverse transcribed with the Uni-12 primer^21^. Each viral gene was amplified using *PfuUltra*® II Fusion HS DNA Polymerase (Stratagene) and gene-specific primers as described^21^. Full-length viral genes were cloned into the plasmid pHW2000^21^,^22^. Mutations were introduced into the pHW2000-AH1-PB2 plasmid using the QuikChange® II Site-Directed Mutagenesis Kit (Stratagene) to generate the K627E, D701N and the double mutations (627E plus 701N). Plasmid pRL-TK (Promega) was renamed as pRluc in this study, which uses thymidine kinase (TK) promoter to drive the constitutive expression of *Renilla* luciferase (Rluc). Reporter plasmid pFluc was constructed by cloning the firefly luciferase (Fluc) gene from the pGL3 vector (Promega) into the RNA polymerase I promoter/terminator cassette. The untranslated regions (UTRs) of the influenza A/WSN/33 NP segment was introduced into the flanking regions of the Fluc gene to produce artificial influenza NP-like RNA segment. All plasmids were confirmed to have the exact sequences as designated.

### Generation of recombinant viruses and virus titration

Recombinant viruses rgAH1-KD (with wild type PB2-627K and 701D), rgAH1-KN (rgAH1-PB2-627K and 701N), rgAH1-ED (rgAH1-PB2-627E and 701D) and rgAH1-EN (rgAH1-PB2-627E and 701N) were generated by co-transfection of the eight reverse-genetically reconstructed plasmids, each carrying a viral gene segment, into 293T/MDCK co-cultured monolayers^22^. The identity of each propagated mutant virus was ascertained by whole genomic sequencing. Viral titrations were determined using MDCK cells, and the TCID_50_ was calculated using the Karber method^23^. Previous reports^9^,^10^,^11^ have shown that H7N9 virus containing PB2-627E and 701D was less virulent than those carrying either PB2-627K-701D or PB2-627E-701K. Because titers of rgAH1-ED stock prepared in MDCK cells were consistently lower than those of the three other mutants by more than ten fold, and rgAH1-ED did not form clear plaques on MDCK cells, rgAH1-ED was not included in the study of virus growth kinetics on A549 cells and the *in vivo* mouse experiment.

### Growth Curves

A549 cells were inoculated with each rescued virus at an M.O.I. of 0.001, and incubated at 33 °C or 37 °C. At time points 0, 12, 24, 36, 48, 60, 72, 84 and 96 hpi, supernatants were collected and TCID_50_ values were determined on MDCK cells.

### Minigenome replication assay

Equal amounts of the reporter plasmid pFluc and internal control plasmid pRluc (0.5 μg each) were co-transfected with expression plasmids encoding PB2, polymerase basic protein 1 (PB1), polymerase acidic protein (PA), and nucleoprotein (NP) genes into 293T cells using the PolyFect (Qiagen) reagent according to the manufacturer’s instructions. Mock transfections were performed with pFluc and pRluc only. Transfected cells were incubated at 33, 35, 37 or 39 °C, respectively. After 24 hours of incubation, supernatants were discarded and the cell extracts were prepared in 120 μl of lysis buffer. Light units were measured using Dual-luciferase Assay Kits (Promega) on a GloMax® 96 Microplate Luminometer (Promega). All results shown are the averages from triplicate experiments with standard deviations (SD).

### Virus infection and transmission in a ferret model

Three six-month-old influenza-free male ferrets (*Mustela putorius furo*, Wuxi Sangosho Co. Ltd.), held in separate cages, were intranasally inoculated with 10^6^ TCID_50_ of AH1 or SH5 viruses in 500 μl of MEM. At 24 hpi, three naïve ferrets (as physical contacts) were introduced to each of the three main cages with the directly inoculated ferrets. Another three were placed in adjacent cages at a 10 cm distance from the main cage, to serve as airborne exposed animals^24^. Nasal washes were collected into 1ml of cold phosphate buffered saline (PBS) on a daily basis and titrated using TCID_50_ assays. When viruses shed by the contact ferrets (either physical or airborne contacts) reached their peak titers, RNAs from the nasal swabs were extracted and subjected to deep sequencing on a Mi-Seq desktop sequencer (Illumina), giving a coverage of 6,000 ∼ 25,000×. The PB2 gene sequences at the 627 and 701 positions were analyzed for their genetic heterogeneity. At 14 dpi, sera were collected from each animal for detection of seroconversion and antibody titers by hemagglutination inhibition (HI) tests.

### Virus infection in the mouse model

Groups of five 8- to 10-week-old specific-pathogen-free (SPF) female C57BL/6 mice (Vital River Laboratories, Beijing) were anesthetized with 0.2 ml of 0.75% pentobarbital sodium and inoculated intranasally with recombinant viruses at the indicated dose (10^1^ ∼ 10^6^ TCID_50_) in a 50 μl volume, or mock inoculated with 50 μl PBS. Body weights and clinical signs of infections were recorded daily. Mice that lost more than 30% of their original weight were euthanized for humane reason and recorded as a fatal infection. At 14 dpi, blood was collected from each of the surviving mice, and serum was separated for determination of antibody titers using HI tests. Mouse median lethal dose (MLD_50_) and 50% infectious dose (MID_50_) were determined using the Karber formula^23^. To determine the virus replication sites, groups of twelve 8- to 10-week old female C57BL/6 mice were intranasally inoculated with 10^3^ or 10^5^ TCID_50_ of the indicated viruses and three mice in each group were euthanized at 1, 3, 5, and 7 dpi. Brain, nasal turbinate, trachea, lung, heart, spleen, kidney, liver, intestinal tract, eyeball, conjunctiva and spinal cord were collected and virus titers were determined by TCID_50_ assays.

### Histological and immunohistochemical examinations

Lung tissues from the infected mice were perfused with 10% neutral buffered formalin and fixed for over 24 h before processing. The tissues were then embedded in paraffin by standard tissue processing procedures, cut into 3 ∼ 4 μm sections and affixed on glass slides. Standard hematoxylin and eosin (H&E, Sigma) staining and immunohistochemical staining of NP antigens in the lung tissues were performed by using a mouse anti-NP monoclonal antibody, kindly provided by Professor Ningshao Xia, the National Institute of Diagnostics and Vaccine Development for Infectious Diseases, Xiamen University, and a goat anti-mouse IgG–biotin conjugated secondary antibody (Calbiochem)^24^.

### Statistical analysis

Differences between experimental groups were evaluated using the Student’s *t* test. A *p*-value* *< 0.05 was considered statistically significant.

## Additional Information

**How to cite this article**: Zhu, W. *et al.* Dual E627K and D701N mutations in the PB2 protein of A(H7N9) influenza virus increased its virulence in mammalian models. *Sci. Rep.* **5**, 14170; doi: 10.1038/srep14170 (2015).

## Figures and Tables

**Table 1:** Virus shed by ferrets inoculated with or exposed to the human H7N9 influenza viruses.

Virus	Infection route	Ferret	Virus shedding			Seroconversion (HI titer at 14 dpi/13 dpe)
			Start day (dpi/dpe)	Duration (days)	Peak titer (log TCID_50_/ml) [dpi/dpe]: PB2-627, 701 residues (percentage)	
A/Anhui/1/2013	Inoculation	In-1	1	6	6.0 [2]: 627K(100%), 701D(100%)	320
		In-2	1	6	6.3 [2]: 627K(100%), 701D(100%)	640
		In-3	1	6	6.0 [2]: 627K(100%), 701D(100%)	320
	Physical contact	PC-1	1	8	7.0 [4]: 627K(100%), 701D(100%)	160
		PC-2	1	6	5.0 [4]: 627K(100%), **D701N(86.8%)**	320
		PC-3	3	4	6.0 [3]: 627K(100%), **D701N(94.2%)**	320
	Airborne exposed	AE-1	—	—	—	40
		AE-2	9	4	6.0 [10]: 627K(100%), 701D(100%)	80
		AE-3	6	6	6.3 [7]: 627K(100%), 701D(100%)	320
A/Shanghai/05/2013	Inoculation	In-1	1	6	5.3 [2]: 627E(100%), 701D(100%)	80
		In-2	1	6	6.3 [2]: 627E(100%), 701D(100%)	160
		In-3	1	6	6.0 [2]: 627E(100%), 701D(100%)	80
	Physical contact	PC-1	—	—	—	40
		PC-2	—	—	—	40
		PC-3	1	8	5.8[4]: 627E(100%), 701D(100%) 5.3[8]: **E627K(30%), D701N(1.1%)**	80
	Airborne exposed	AE-1	—	—	—	—
		AE-2	—	—	—	—
		AE-3	—	—	—	—

-: virus or HI antibody not detected. dpi/dpe: days post-inoculation or post-exposure. HI titer: heamagglutination inhibition titer. In-#: ferret intranasally inoculated with the virus; PC-#: ferret exposed to the infected animals via physical contact; AE-#: ferret exposed to the infected animals via airborne exposure.

**Table 2:** Seroconversions of the C57BL/6 mice inoculated with recombinant H7N9 viruses.

Virus	Inoculation dose (log TCID_50_/50μl)	HI titer					MID_50_ (log TCID_50_)
		#1	#2	#3	#4	#5	
**rgAH1-KD**	6	ND	ND	ND	ND	ND	1.1
	5	160	ND	ND	ND	160	
	4	320	320	160	160	ND	
	3	160	160	160	160	160	
	2	160	160	80	80	<10	
	1	160	160	160	<10	<10	
**rgAH1-KN**	6	ND	ND	ND	ND	ND	0.9
	5	160	ND	ND	ND	ND	
	4	ND	ND	ND	ND	ND	
	3	160	160	ND	ND	320	
	2	320	160	160	160	20	
	1	160	160	160	160	10	
**rgAH1-EN**	6	320	160	160	40	ND	2.5
	5	640	160	160	80	80	
	4	320	160	160	160	160	
	3	160	160	80	40	10	
	2	160	<10	<10	<10	<10	
	1	<10	<10	<10	<10	<10	
**PBS**		<10	<10	<10	<10	<10	

Serum collected at 14 days post-inoculation from each mouse (#1 to #5, respectively) was tested for heamagglutination inhibition (HI) titers against the A/Anhui/1/2013 (H7N9) virus. Median infectious dose (MID50) was determined using the Karber Method^23^. ND: Not determined due to death of the animal (treated as positive for determination of the MID_50_). HI titer <40 was regarded as negative for seroconversion.
